# The Fifth New York District Dental Society

**Published:** 1870-07

**Authors:** 


### Proceedings of Societies.

### THE FIFTH NEW YORK DISTRICT DENTAL SOCIETY.

The second annual meeting of the Fifth New York District Dental Society, was held at Watertown, New York, on Tuesday, June 14, 1870. Dr. S. B. Palmer, of Syracuse, President of the Society, delivered the annual address, and the following officers were duly elected for the ensuing year: President, A. N. Priest, Utica; Vice President, J. C. House, Lowville; Secretary, Chas. Barnes, Syracuse; Corresponding Secretary, A. S Roberts, Rome; Treasurer, P. Harris, Skaneateles; Board of Censors, L. W. Rogers, Utica; S. B. Palmer, Syracuse; S. M. Robinson, Watertown. Dr. Palmer was also chosen permanent delegate of the society.

Several new members were added to the society, which may now be considered as well organized and in good working order.

The title of the annual address, was: The individuality and self-reliance of Dentists, and was as follows:

MR. PRESIDENT AND MEMBERS OF THE FIFTH DISTRICT DENTAL SOCIETY:—Permit me upon this occasion to thank you for the honor conferred in calling me to the chair; also for the patience manifested during our sessions. I would not embrace such an opportunity merely to retain the floor, but there are some general topics which we believe demand discussion, and I therefore intend to briefly mention a few, and if possible, will solve the mystery why certain materials and methods of practice are so extolled and so successful with some, and yet are denounced as worthless by others.

The progress and changes in Dentistry are so rapid, that constant study and investigation become a duty we owe to those who confide in our practice, and he who neglects the means of procuring all the knowledge within his reach, under the plea of business, is selfish, and unfaithful to that class of patients who are seeking the highest skill.

Time is necessary to determine the value of a new discovery; every year new discoveries confront us; at meetings like this we should investigate and put them on trial.

I know of no profession or calling that demands such individuality and self-reliance, as Dentistry. When the young artist has finished his classical studies, his education is not considered complete, without the advantages of a European tour, and the opportunities it affords to receive the highest ideas to be gained from inspecting the works of the old masters; but Dentists are like Napoleon's soldiers, who, when landed upon the shores of a country where prompt and decisive work was to be done, saw by command of their leader, their boats burned and every means of retreat cut off. Under such circumstances, with Napoleon at their head, victory was certain.

Our professional course too is onward.

We are now only entering upon the third year from the date of the legislative action, regulating Dentistry in our State. The result of that action will inevitably divide Dentists into two classes, viz: Those willing to teach and anxious to learn, and those who profess to know all, yet hesitate to communicate their knowledge. From the latter we look for new members to fill these vacant seats.

Ask the Dentists in this district, not present, the cause of their absence, and the usual answer "crowded engagements" will be assigned. There is haste that makes waste. Artists understand this. A few days ago, I saw a celebrated scene painter engaged upon a large drop-curtain, which must be finished by a certain day. I found him quietly seated at the rear of the hall, far from brush and canvass, reviewing the portion of the work accomplished, and designing plans for future action. So to-day, away from office and patients, we may be doing our work as speedily and more effectually than those who remain at home.

I have thought it profitable to mention some of the modern discoveries and forms of materials that have lately been introduced and used by some of the members present, in hope to gain knowledge of their real worth in general practice.

We can not at once adopt the practice of another, however superior it may be, if it requires the use of different instruments and change of manipulation, any more than we could expect to speak French, by listening to a lecture upon the advantages of understanding the French language. This we regard as the diverging point respecting discoveries of real merit. There are many noble minds in our profession, and we are proud of them. They possess education, skill, practical ability, and have position to command respect and admiration. With such advantages it is but natural that they are looked upon as leaders, yet what such minds have mastered, have been wrought out by the same process of thought and experiment that each of us must adopt to gain the same ends. I will sit at the feet of him who will teach me.

In the science of Dentistry, each operator in his practice must govern himself in accordance with his abilities, education and appreciation, but he makes a sad mistake who attempts to leap the chasm of experiment and follow a leader who has spent perhaps years to accomplish success. It is well to receive new ideas, but we can introduce them only as we can educate our hands to work them out. When we can do this, though our practice is changed, it is still ours. We are not followers, but fellow travelers.

A few years since, Dr. Atkinson introduced the mallet in plugging teeth. Convinced as he was of its value, skilled as he was in its use, his very nature prompted him to extend its advantages to others. No sooner was the announcement made public, than the hero was followed by a mallet brigade, who did fearful work in their first assault on frail sensitive dentine. To condemn the mallet for this misdirected application, would be as inconsistent as to condemn systematic warfare because the Fenians failed to conquer Canada. To-day the mallet in tooth filling is a symbol of solidity and success. In its introduction we see that all are not followers. Some of our first class operators have never yet attempted its use; they show their individuality by so doing, and recognize the fact that in the use of a new instrument each must learn for himself. The same general rule applies to the use of new materials, as well as the perception to determine pathological conditions. About three years ago, oxy-chloride of zinc was introduced as an article for capping exposed tooth pulps. At Niagara Falls, warm earnest discussions followed. Its value was set forth by our best instructed Dentists, and was substantiated by successful experiments, while one member upon the opposite side manifested surprise at such different results from the use of the same article in different hands; he had used it in a similar way as the best agent for devitalizing pulps. Last summer, at Saratoga, many new converts gave testimony in its favor. I am convinced by experiments that it has merit, and shall certainly continue my investigations until I ascertain my abilities to use it.

Plastic gold, too, has been presented to the profession as the one thing needful in filling teeth. Experiments in my own hand have been attended with results so unlike and uncertain, that I conclude it not worth further exertion to perfect myself in its use. Yet I can not speak for the success of others.

Again, and later, we have the results of experiments laid before us in the use of heavy foil. We all know how the idea at first strikes us whose practice has been with Nos. 3 and 4 foil.

Gentlemen, opinions amount to little. I have charity to award the success and superiority claimed by those educated in its use; but for me to follow and expect at first to achieve the same brilliant success, would be to condemn heavy foil. I must tread the path they have passed over if I hope to attain the same ends. It may be worth the time and expense to me, it may not, that I must determine for myself.

The rubber dam also commands our notice. I have not yet been able to realize any great advantage from its use, yet the rubber dam has merit, and we should not follow the example of one who said: "I have tried it," and to give you the result of his experiment, in as short and polite language as possible, you have only to read the name backwards.

Gentlemen, let us be slow to condemn, earnest in experimenting, and cautious in adopting a practice until familiarity has made it our own. Let us do honor to our superiors, as teachers and benefactors, but follow no man as leader.

Just in proportion as every man regards himself and his field of labor, a little universe, alone ruled over and guided by his own soul—(which is part of and second only to its creator)—will he possess originality and individuality. When we live up to this heaven born privilege, we will be no longer followers, but doers in the noble work in which we are engaged, capable of willing and acting for ourselves.

